# Guillain-Barré-like Syndrome, as a Rare Presentation of Adult T-cell Leukemia-Lymphoma (ATLL): A Case Report

**Published:** 2012-08-30

**Authors:** Payam Sasannejad, Mahmoud Reza Azarpazhooh, Hossein Rahimi, Amir Moghaddam Ahmadi, Ali Mellat Ardakani, Hamid Reza Saber

**Affiliations:** 1 Department of Neurology, Mashhad University of Medical Sciences, Mashhad, IR Iran; 2 Department of Hematology, Mashhad University of Medical Sciences, Mashhad, IR Iran; 3 Faculty of Medicine, Mashhad University of Medical Sciences, Mashhad, IR Iran

**Keywords:** Guillain-Barré Syndrome, Human T-lymphotropic virus 1, Leukemia-Lymphoma, Adult T-Cell

## Abstract

We report a 21-year-old woman who was admitted because of unilateral facial paresis and then developed progressive ascending flaccid tetraparesis with generalized areflexia. Electrodiagnostic studies revealed acute motor axonal polyradiculoneuropathy (AMAN type of Guillain-Barré Syndrome). Further evaluations revealed severe leukocytosis, increased erythrocyte sedimentation rate (ESR), increased protein content and presence of a few lymphocytes in cerebrospinal fluid (CSF), and then presence of human T-cell lymphotropic virus type 1 (HTLV-I) in serum and CSF. Finally, biopsy of the enlarged lymph nodes resulted in the diagnosis of Adult T-cell Leukemia-Lymphoma. The HTLV-1 has been endemic to certain parts of Iran like Khorasan province in the northeast since 1985 with 2.3% prevalence rate of infection. Thus, some rare neurologic complications occasionally occur in this area as a result of being infected with HTLV-1.

## 1. Introduction

Adult T-cell Leukemia/Lymphoma (ATLL) is a non-Hodgkin T-cell Lymphoma that is linked to infection by the human T-cell lymphotropic virus type 1 (HTLV-1). It is characterized by skin and lung involvement, hepatosplenomegaly, lymphadenopathy and hypercalcemia. The clinical course has a terminal end in most of the acute cases. It was first described by Uchiyama and co-workers in 1977 in southwestern Japan [1].

The HTLV-1 has been endemic to certain parts of Iran like Khorasan province in the northeast since 1985 with 2.3% prevalence rate of infection [2]. Its prevalence in Mashhad, the capital city of Khorasan state, has been estimated 3% in the whole population [2], and 0.7 % to 1.16% in blood donors [3]. Thus, some rare neurologic complications occasionally occur in these areas as a result of being infected with HTLV-1.

## 2. Case Report

A 21-year-old woman was admitted to Qaem Hospital neurology ward in Mashhad, having left facial paresis at onset. During the period of hospitalization, she had pain and paresthesia in the lower limbs along with an insidious weakness which was rapidly progressing in an ascending fashion. Neurological examination revealed left peripheral facial paresis, flaccid tetraparesis, areflexia and also impairment of deep sensations in the lower limbs. The primary laboratory tests showed leukocytosis (33000/microliter), anemia and hypercalcemia. Evaluation of the cerebrospinal fluid demonstrated 10 lymphocytes/HPF and increased protein content (239 mg/dl). Electrodiagnostic studies revealed predominantly axonal, motor polyradiculoneuropathy (AMAN type of GBS); therefore, the patient was diagnosed with Guillain-Barré syndrome and her treatment with intravenous human immunoglobulin (IVIG) was started.

Through further evaluations, we found elevated ESR (95mm/hr) and liver enzymes (SGOT = 198 and SGPT = 265 mg/dl). In addition, the evaluation of the PCR (polymerase chain reaction) revealed the presence of HTLV-1 in serum and CSF. Other tests for assessing collagen vascular disorders, vasculitides, thyroid dysfunction, and other infections such as tuberculosis, brucellosis, syphilis, HIV infection, etc. were negative.

**Figure 1:**
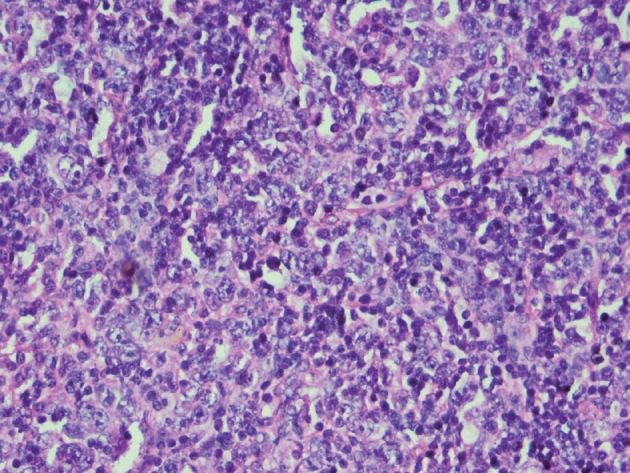
Proliferation of tumor cells (Immunoblasts) with prominent nuclei and abundant mitosis mixed with lymphocyte cells and eosinophils and some Histiocytes and postcapillary venuls proliferation. Immunohistochemical analysis of the tumor cells revealed that they were LCA and CD3 positive; CD15 and CD20 negative.

In abdominal ultrasonography, periaortic and mesenteric lymphadenopathy was found, but the size of liver and spleen was normal. Bone marrow aspiration was accomplished that was normal, but biopsy of a cervical lymph node confirmed T-cell Non-Hodgkin Lymphoma (NHL) (Figure 1). Immunohistochemical studies led to the diagnosis of ATLL. Regarding the diagnosis of ATLL, the patient underwent a course of chemotherapy but despite extensive therapeutic and supportive care in the intensive care unit (ICU), she passed away after 3 weeks due to the complications.

## 3. Discussion

Guillain-Barré syndrome is an inflammatory polyradiculoneuropathy. In approximately two third of the cases, symptoms often begin after an acute infectious process, immunization, or surgery [4][5]. The syndrome has also been reported in association with some malignancies including lymphoma particularly the Hodgkin’s Lymphoma (HL), small cell lung cancer and leukemia [6][7][8], but there is not any documented report of GBS as the primary presentation of ATLL. Adult T-cell leukemia/lymphoma (ATLL) is a rare and aggressive T-cell lymphoma that is associated with HTLV-1 infection [1]. It is characterized by the presence of malignant T-cells in the peripheral blood, that have highly indented or lobulated nuclei. Clinically, ATLL may present as leukemia, lymphoma, hypercalcemia, and tumor infiltrates of the skin or lungs and hepatosplenomegaly.

HTLV-I infection is endemic in tropical areas such as South America, Central Africa, Southern Japan, and Middle East like Khorasan province of Iran [9]. Its prevalence in Mashhad, the capital city of Khorasan province, has been estimated 3% in the whole population, and 0.7% to 1.16% in blood donors [1][2]. The main manifestations of HTLV-I infection are neurologic and hematologic (such as ATLL) disorders. Its main neurologic manifestation is HTLV-I Associated Myelopathy (HAM), but other disorders such as peripheral neuropathy, myopathy, peripheral facial paresis, etc. have been reported too [9].

Healthy relatives of patients with ATLL have an especially high incidence of sero-positivity [1]. It is remarkable that 4 of the 7 members of our patient’s immediate family were sero-positive for HTLV-1 infection. This case describes an extremely rare presentation and clinical course of ATLL with a Guillain-Barre’-like syndrome in a young woman. Thus, in patients with manifestations resembling GBS, it is important to consider all the possible etiologies that can lead to this syndrome, and especially this is of value for diseases that are rare, but endemic, in specific geographic regions.
